# The use of neural‐derived extracellular vesicles as a biomarker tool of neuropathology (with focus on Alzheimer’s disease and Lewy body dementia)

**DOI:** 10.1002/alz.092728

**Published:** 2025-01-09

**Authors:** Millie Sander, Esra Bozbas, Clive G Ballard

**Affiliations:** ^1^ University of Exeter, Exeter UK; ^2^ University of Exeter, Exeter, Devon UK

## Abstract

**Background:**

Establishing novel biomarkers to detect of Alzheimer’s disease (AD) and Lewy body dementia (LBD) neuropathology is integral to support early diagnosis and the differentiation between dementia subtypes. Neural‐derived extracellular vesicles (NDEVs) – released by neural cells into circulation – provide promise in this regard. NDEV cargo, such as p‐T181‐tau and Aβ‐42, have previously been shown to be associated with cognitive decline and disease progression among AD patients (Winston et al, 2016; Kapogiannis et al, 2019). Here, we assess various protocols for the isolation of NDEVs, with the aim of establishing both AD and LBD NDEV profiles.

**Method:**

Plasma samples are being collected from individuals with AD, LBD, MCI and healthy controls. Three EV isolation techniques – differential ultracentrifugation, size exclusion chromatography, and ExoQuick – are being compared to assess the presence of NDEVs (via western blot and transmission electron microscopy), as well as their size and yield (via nanoparticle track analysis). The enrichment of EVs of neural origin using L1CAM and NCAM will be assessed, to address conflict and doubt in the field.

**Result:**

The data for this study is currently being collected, and results will be available prior to the conference.

**Conclusion:**

NDEVs present a promising tool for detecting and monitoring neuropathology during dementias such as AD and LBD. The optimal isolation technique for NDEVs is still controversial, and so this work will address said problem.

